# Antibiotic Resistance Patterns of Bacteria Isolated from Canine Skin and Ear Infections in Serbia

**DOI:** 10.3390/antibiotics15010021

**Published:** 2025-12-23

**Authors:** Isidora Prošić, Branislav Vejnović, Dušan Mišić, Andrea Radalj, Aleksandar Nikšić, Ksenija Aksentijević, Marina Radojičić, Vladimir Gajdov, Milica Ilić, Natalija Milčić Matić, Dejan Krnjaić

**Affiliations:** 1 Department of Microbiology, Faculty of Veterinary Medicine, University of Belgrade, Bul. Oslobodjenja 18, 11000 Belgrade, Serbia; isidora.prosic@vet.bg.ac.rs (I.P.); aleksandar.niksic@vet.bg.ac.rs (A.N.); ksenija@vet.bg.ac.rs (K.A.); marina.radojicic@vet.bg.ac.rs (M.R.); milica.ilic429@gmail.com (M.I.); dejan.krnjaic@vet.bg.ac.rs (D.K.); 2 Department of Economics and Statistics, Faculty of Veterinary Medicine, University of Belgrade, Bul. Oslobodjenja 18, 11000 Belgrade, Serbia; branislavv@vet.bg.ac.rs; 3 Department of Functional Food Products Development, Faculty of Biotechnology and Food Sciences, Wrocław University of Environmental and Life Sciences, 51-630 Wrocław, Poland; dusan.misic@upwr.edu.pl; 4 Scientific Veterinary Institute “Novi Sad”, 21113 Novi Sad, Serbia; vladimir.g@niv.ns.ac.rs; 5 VDC Veterinary Clinic, Jovana Rajića 5, 11000 Belgrade, Serbia; 6 Department of Equine, Small Animal, Poultry and Wild Animal Diseases, Faculty of Veterinary Medicine, University of Belgrade, Bul. Oslobodjenja 18, 11000 Belgrade, Serbia; natalija.milcic@vet.bg.ac.rs

**Keywords:** antibiotics, antimicrobial resistance, dogs, florfenicol, multidrug resistance, otitis externa, skin infections

## Abstract

**Background**: Canine skin and ear infections are common in small-animal practice and increasingly complicated by multidrug resistance (MDR), yet data from Serbia are limited. This study aimed to describe the bacterial etiology and antimicrobial resistance patterns in canine otitis externa and pyoderma. **Methods**: We retrospectively reviewed laboratory records from the Clinical Bacteriology and Mycology Laboratory, Faculty of Veterinary Medicine, University of Belgrade (January 2017–August 2024). A total of 422 non-invasive swabs from clinically ill dogs were included (ears: *n* = 210; skin: *n* = 212). Bacterial identification used conventional methods and commercial systems, and disk-diffusion susceptibility testing followed CLSI/EUCAST guidance. Methicillin resistance in staphylococci was assessed by cefoxitin/oxacillin screening; MRSA was confirmed by PCR and PBP2a detection. Resistance trends were compared between 2017–2020 and 2021–2024. **Results**: The leading pathogens were *Staphylococcus pseudintermedius* (ears 48.1%; skin 79.7%) and *Pseudomonas aeruginosa* (ears 29.1%; skin 7.6%). Staphylococci showed high resistance to macrolides, clindamycin, tetracycline, and first-line β-lactams (amoxicillin–clavulanate, cephalexin), with the highest susceptibilities to amikacin, florfenicol, and rifampicin. *P. aeruginosa* remained most susceptible to amikacin, polymyxin B, and imipenem. Between the two periods, *S. pseudintermedius* resistance increased to amikacin, fusidic acid, and cephalexin, while resistance to florfenicol decreased. *P. aeruginosa* resistance to imipenem increased. The prevalence of methicillin-resistant *S. pseudintermedius* (MRSP) was 27.4% (74/270). MDR *S. pseudintermedius* and MDR *P. aeruginosa* were identified in 38.5% and 53.3% of isolates, respectively. One isolate of each species was resistant to all tested drugs. **Conclusions**: These findings confirm high levels of antimicrobial resistance in major canine skin and ear pathogens and emphasize the need for susceptibility-based therapy, rational antimicrobial use, and ongoing surveillance in small-animal practice.

## 1. Introduction

Bacterial skin and ear infections are among the most common conditions in small animal veterinary practices [1,2]. Skin infections may present with mild erythema and pruritus or progress to deep, painful, or ulcerative lesions, while otitis externa (OE) may range from mild erythema and ceruminous discharge to chronic, painful, or proliferative disease. These infections are typically caused by the skin and ear microbiota of dogs and cats and most often occur as secondary complications. Moderate to severe or recurrent cases are usually triggered by stress, dietary changes, or underlying pathological conditions, including atopic dermatitis, allergies, endocrinopathies, ectoparasites, foreign bodies, and anatomical abnormalities [1,2,3]. Such events, combined with breed susceptibility and other predisposing factors, create favorable conditions for colonization by opportunistic pathogens [1,2].

The most commonly isolated causative agents in canine skin and ear infections are *S. pseudintermedius*, *S. aureus*, *Streptococcus canis*, *P. aeruginosa*, *Escherichia coli*, and *Proteus* spp., with *Malassezia pachydermatis* frequently found in cases of OE [1]. These infections not only cause significant discomfort and pain in affected animals [4], but they also pose a public health concern [1,2]. The increase in the global companion animal population, especially of dogs, further amplifies this risk, as dogs can serve as reservoirs for antimicrobial resistance (AMR) determinants, transmissible through direct or indirect contact [5]. The close relationship between pets and their owners may favor such transmission [1].

The development of AMR in bacterial pathogens and the emergence of multidrug-resistant (MDR) microorganisms have profound implications for both veterinary and public health. Dogs carrying MDR pathogens are at an increased risk of failed treatment regimens, with severe cases requiring last-resort surgical interventions [2]. Despite these challenges, empirical antibiotic prescription without prior susceptibility testing remains common in veterinary medicine, particularly in developing countries such as Serbia, creating conditions that favor the selection and dissemination of resistant strains [1].

Given the significance of MDR bacterial strains, it is crucial to maintain updated knowledge about the pathogens implicated in canine skin and ear infections and their resistance patterns. Regular monitoring and temporal analysis are essential for designing effective control strategies, improving treatment outcomes, and enabling evidence-based therapies. Therefore, the aim of this study is to highlight the importance of routine microbiological diagnostics by analyzing data collected over eight years at the Laboratory for Clinical Bacteriology and Mycology, Faculty of Veterinary Medicine (FVM), University of Belgrade. Additionally, it evaluates antibiotic resistance profiles, highlighting the high prevalence of MDR strains and helps inform appropriate antibiotic use and control measures.

## 2. Results

In this retrospective study, a total of 422 samples from cases of canine ear (*n* = 210) and skin (*n* = 212) infections were analyzed. The number of bacterial skin infections was nearly identical to the number of OE cases.

In canine ear samples, *S. pseudintermedius* was the most commonly identified bacterium (101/210; 48.10%), followed by *P. aeruginosa* (61/210; 29.05%). *S. aureus* was isolated from 18 swabs (18/210; 8.57%), and other staphylococci were found in 16 swabs (16/210; 7.62%). Among the other staphylococci, the following species were identified: *S. coagulans* from 1 swab (0.48%), *S. haemolyticus* from 3 swabs (1.43%), and *S. epidermidis* from 12 swabs (5.71%). *Streptococcus* Lancefield group G (presumptive *S. canis*) was isolated from 23 swabs (23/210; 10.95%). *Proteus* spp. was isolated from 39 samples (39/210; 18.57%), comprising *P. vulgaris* in 9 swabs (9/210; 4.29%) and *P. mirabilis* in 30 swabs (30/210; 14.29%). The prevalence of Enterobacterales was 4.29% (9/210) and the following species were identified: *Escherichia coli* in 4 cases (4/210; 1.90%), *Klebsiella pneumoniae* in 4 swabs (4/210; 1.90%), and *Serratia marcescens* in 1 swab (1/210; 0.48%). Other bacteria identified included *Corynebacterium* sp. in 17 swabs (17/210; 8.10%), and a single isolate of *Pseudomonas* sp. (other than *P. aeruginosa*) (0.48%). These bacteria were identified as causative agents of OE in dogs (Table 1). Of the 210 ear swabs, 69 (32.86%) showed mixed bacterial infections caused by two or more bacteria.

Regarding canine skin samples, *S. pseudintermedius* was the most commonly identified bacterium (169/212; 79.72%), followed by *S. aureus* (22/212; 10.38%). Other staphylococci were isolated from 10 swabs (10/212; 4.72%) represented by the following species: *S. coagulans* (4/212; 1.89%), *S. epidermidis* (4/212; 1.89%), and *S. haemolyticus* (2/212; 0.94%). Beta-hemolytic *Streptococcus* Lancefield group G was isolated from 18 swabs (18/212; 8.49%). *P. aeruginosa* was isolated from 16 swabs (16/212; 7.55%). *Proteus* spp. was isolated from 18 samples (18/212; 8.49%), comprising *P. vulgaris* in 8 swabs (8/212; 3.77%) and *P. mirabilis* in 10 swabs (10/212; 4.72%). Among the Enterobacterales, *E. coli* was the only isolated species in 11 cases (11/212; 5.19%). Other identified bacteria included *Corynebacterium* sp. in 3 swabs (3/212; 1.42%) and *Pseudomonas* sp. (other than *P. aeruginosa*) in 2 swabs (2/212; 0.94%) (Table 1). Compared to cases of OE, mixed bacterial infections were less common in skin samples, with 52 out of 212 skin swabs (24.53%) showing mixed infections caused by two or rarely three bacteria.

**Figure 1:**
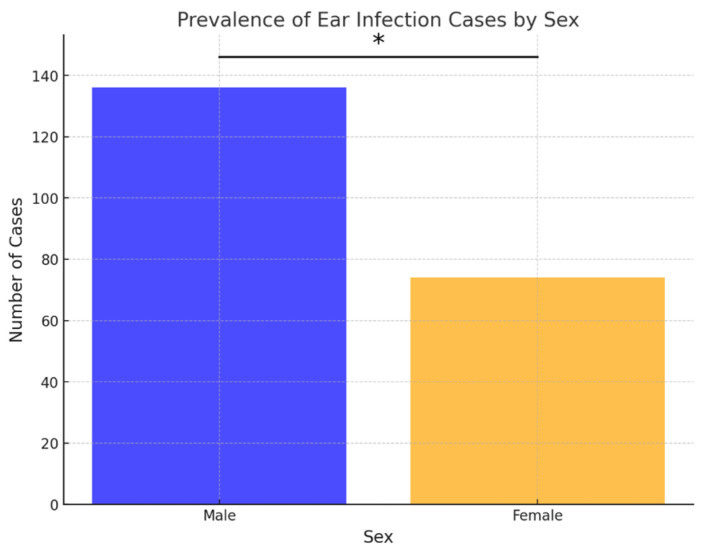
Prevalence of ear infection cases by sex. Asterisk indicates a statistically significant difference between sexes (Fisher’s exact test, *p* < 0.05).

**Figure 2:**
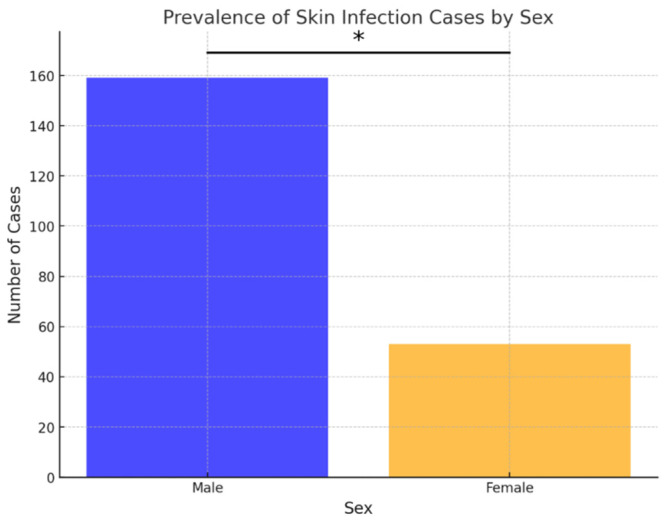
Prevalence of skin infection cases by sex. Asterisk indicates a statistically significant difference between sexes (Fisher’s exact test, *p* < 0.05).

Out of the 210 ear samples, 136 (64.76%) were from males, and 74 (35.24%) were from females. Similarly, in cases of skin infections, 159 out of 212 samples (75%) were from males, and 53 (25%) were from females. Over the examined 8-year period, the frequency of both skin and ear infections was statistically higher in males compared to females (*p* < 0.05) (Figure 1 and Figure 2).

**Figure 3:**
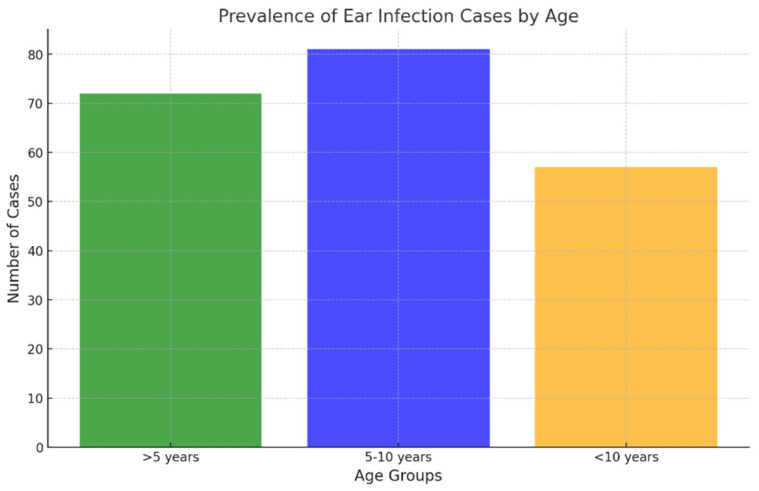
Prevalence of ear infection cases by age.

**Figure 4:**
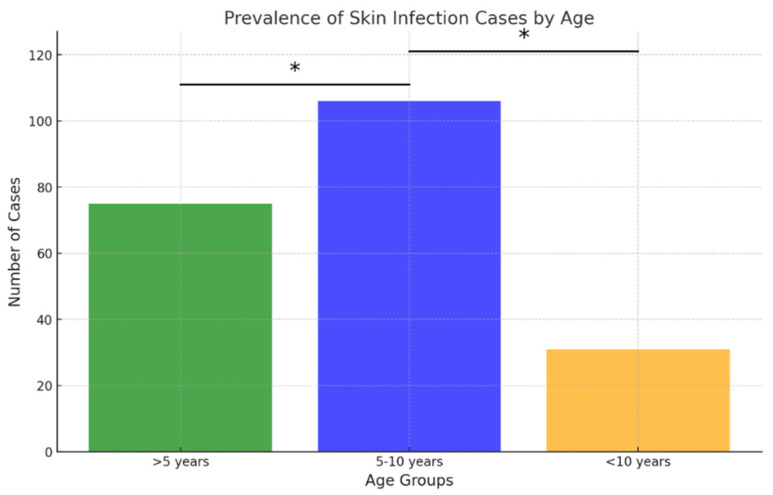
Prevalence of skin infection cases by age. Asterisk indicates a statistically significant difference between age groups (Fisher’s exact test, *p* < 0.05).

Regarding age distribution, the 5–10-year age group accounted for the largest proportion of samples (81/210; 38.57% of ear samples and 106/212; 50% of skin samples), followed closely by dogs younger than 5 years (72/210; 34.29% of ear samples and 75/212; 35.38% of skin samples) (Figure 3 and Figure 4). The 5–10 age group showed statistically higher infection rates for skin infections (*p* < 0.05), while no statistically significant differences in infection rates were observed for ear infections.

In cases of OE, a total of 47 different dog breeds were identified. The most frequently observed breed was the West Highland White Terrier (*n* = 32; 15.24%), followed by the Labrador Retriever (*n* = 14; 6.67%), English Bulldog and English Cocker Spaniel (*n* = 12 each; 5.71%), Golden Retriever and mixed breeds (*n* = 10 each; 4.76%), Shar Pei and Pug (*n* = 9 each; 4.29%), and Bichon Frisé, Poodle, and French Bulldog (*n* = 8 each; 3.81%). In cases of skin infections, a total of 35 different dog breeds were identified. The most frequently observed breed was the American Staffordshire Terrier (*n* = 37; 17.45%), followed by the Bull Terrier (*n* = 23; 10.85%), English Bulldog (*n* = 20; 9.43%), Doberman Pinscher (*n* = 16; 7.55%), German Shepherd and mixed breeds (*n* = 13 each; 6.13%), French Bulldog (*n* = 10; 4.72%), and West Highland White Terrier and Boxer (*n =* 8 each; 3.77%). Other breeds were represented in smaller numbers (fewer than 8 individuals).

### 2.1. Overall Antimicrobial Susceptibility Profile

**Figure 5:**
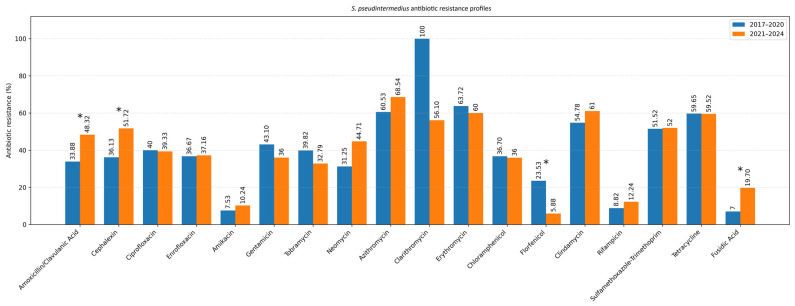
Resistance profile of *S. pseudintermedius* (Comparison of the 2017–2020 and 2021–2024 periods). Asterisk indicates a statistically significant difference (Fisher’s exact test, *p* < 0.05).

The antimicrobial susceptibility results are presented separately for six examined bacterial groups and are summarized in Table 2. The following results represent the overall antibiotic susceptibility profile of all isolates collected during the entire 2017–2024 study period. Pairwise comparisons were performed to evaluate differences in susceptibility proportions among antibiotics within the same timeframe. Temporal trends in resistance between the two study periods (2017–2020 vs. 2021–2024) are presented separately in Figure 5.

Across staphylococcal species, the highest resistance rates were observed in *Staphylococcus pseudintermedius* for the macrolides azithromycin (130/203; 64.0%) and erythromycin (96/153; 62.7%)—and for tetracycline (93/156; 59.6%), whereas the greatest susceptibility was recorded for florfenicol (157/170; 92.4%) and amikacin (235/259; 90.7%), with similarly high values for rifampicin (148/166; 89.2%) and fusidic acid (146/166; 88.0%). Pairwise comparisons confirmed that amikacin had higher susceptible fractions than gentamicin (162/266; 60.9%; *p* < 0.0001), tobramycin (109/174; 62.6%; *p* < 0.0001) and neomycin (58/101; 57.4%; *p* < 0.0001), while florfenicol showed higher susceptibility than β-lactams and fluoroquinolones (vs amoxicillin/clavulanate 157/270; 58.1%; *p* < 0.0001; vs. ciprofloxacin 163/270; 60.4%; *p* < 0.0001) and macrolides and tetracycline (all *p* < 0.0001). Chloramphenicol susceptibility rates (167/262; 63.7%) were significantly higher than those of clarithromycin (18/43; 41.9%; *p* = 0.0001), erythromycin (*p* < 0.0001), clindamycin (91/215; 42.3%; *p* < 0.0001), sulfamethoxazole–trimethoprim (113/234; 48.3%; *p* = 0.0006) and tetracycline (*p* < 0.0001). In Staphylococcus aureus, amikacin had higher susceptibility rates than β-lactams (*p* < 0.0001; *p* = 0.0002), fluoroquinolones (both *p* = 0.0025), macrolides (*p* < 0.0001–0.0003), SXT (*p* = 0.0026) and tetracycline (*p* < 0.0001), while florfenicol showed higher susceptibility than β-lactams (both *p* < 0.0001), fluoroquinolones (*p* = 0.0002), macrolides (all *p* < 0.0001), SXT (*p* < 0.0001), tetracycline (*p* = 0.0217) and chloramphenicol (*p* = 0.01). Chloramphenicol had higher susceptibility than amoxicillin/clavulanate (*p* = 0.0193), cephalexin (*p* = 0.0438), macrolides (*p* = 0.0066–0.0253), SXT (*p* = 0.0006) and tetracycline (*p* = 0.0133). In other staphylococci, florfenicol (23/24; 95.8%) and amikacin (24/26; 92.3%) were most active, and pairwise analysis showed that amikacin exceeded gentamicin (*p* = 0.0385), azithromycin (*p* = 0.0065) and SXT (*p* = 0.0161), while florfenicol exceeded cephalexin (*p* = 0.0479), azithromycin (*p* = 0.0026), SXT (*p* = 0.0037) and clindamycin (*p* = 0.0258). Among β-hemolytic Streptococcus group G, the highest susceptibility was observed for florfenicol (27/28; 96.4%), chloramphenicol (38/41; 92.7%), cephalexin (36/39; 92.3%), rifampicin (12/13; 92.3%) and amoxicillin/clavulanate (37/41; 90.2%), while the lowest occurred for ciprofloxacin (46.3%), enrofloxacin (48.8%), SXT (34.4%), tetracycline (25.0%) and fusidic acid (35.7%). For this species, β-lactams had higher susceptibility than fluoroquinolones (all *p* < 0.0001) and clindamycin (*p* = 0.0390; *p* = 0.0291), while chloramphenicol and florfenicol exceeded fluoroquinolones and all low-performing classes (all *p* < 0.0001); chloramphenicol also exceeded erythromycin (*p* = 0.0385) and clindamycin (*p* = 0.0153). In *Pseudomonas aeruginosa*, the highest susceptibility was observed for amikacin (69/76; 90.8%), polymyxin B (60/67; 89.6%), imipenem (48/54; 88.9%), enrofloxacin (83.1%), ciprofloxacin (81.8%), gentamicin (80.7%) and tobramycin (80.6%), while florfenicol was significantly lower (29/59; 49.2%), with pairwise comparisons confirming its lower susceptibility rates versus ciprofloxacin (*p* = 0.0054), enrofloxacin (*p* = 0.0029), amikacin (*p* < 0.0001), gentamicin (*p* = 0.0068), tobramycin (*p* = 0.0091), polymyxin B (*p* = 0.0003) and imipenem (*p* = 0.0008). In *Proteus mirabilis*, imipenem showed the highest susceptibility (26/26; 100%), followed by amikacin (92.5%), gentamicin (86.0%), ciprofloxacin (84.2%), enrofloxacin (84.2%), tobramycin (81.6%), neomycin (77.5%) and cephalexin (64.8%), whereas florfenicol (65.3%), amoxicillin/clavulanate (63.2%), chloramphenicol (59.6%), SXT (51.1%) and tetracycline (48.9%) showed lower values; imipenem exceeded all lower-performing drugs (*p* = 0.0003–0.0232), and amikacin exceeded β-lactams, chloramphenicol, florfenicol, SXT and tetracycline (*p* = 0.0003–0.0075).

### 2.2. Temporal Trends in Antimicrobial Resistance (2017–2024)

**Figure 6:**
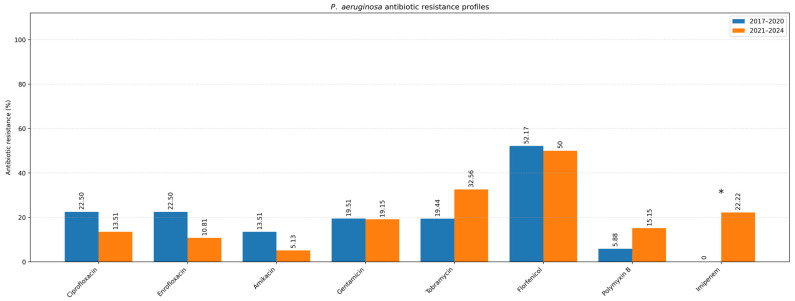
Resistance profile of *P. aeruginosa* (Comparison of the 2017–2020 and 2021–2024 periods). Asterisk indicates a statistically significant difference (Fisher’s exact test, *p* < 0.05).

**Figure 7:**
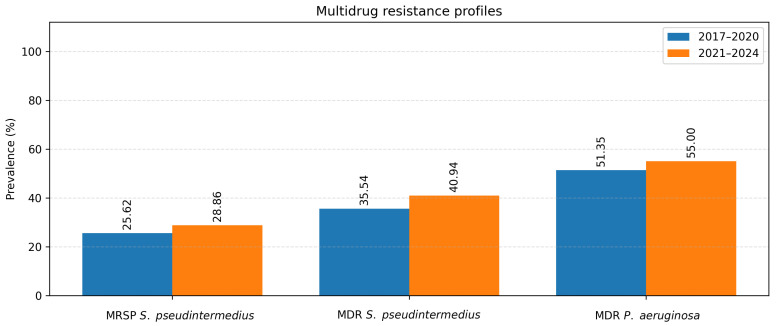
MRSP, MDR *S. pseudintermedius* and MDR *P. aeruginosa*.

To evaluate temporal changes in antimicrobial resistance, data were compared between two study periods: 2017–2020 and 2021–2024 (Table 3). The analysis focused on the two most common pathogens isolated from canine ear and skin infections, *S. pseudintermedius* and *P. aeruginosa*. For *S. pseudintermedius*, resistance to several antibiotics significantly increased in the later period. Notably, resistance to amoxicillin–clavulanic acid and fusidic acid showed a statistically significant increase (*p* < 0.05), observed among both methicillin-resistant and methicillin-susceptible isolates. A similar upward trend was noted for cephalexin (*p* < 0.05), corresponding to the rising proportion of methicillin-resistant isolates. In contrast, a statistically significant decrease in resistance to florfenicol was observed (*p* < 0.05) (Figure 5). Resistance to macrolides (azithromycin, erythromycin, clarithromycin) and tetracycline remained high but relatively stable across both periods, indicating sustained selective pressure within these antimicrobial classes. For *P. aeruginosa*, a significant increase in resistance to imipenem was recorded in 2021–2024 compared to 2017–2020 (*p* < 0.05) (Figure 6). Resistance levels to other antipseudomonal agents such as amikacin and polymyxin B remained largely unchanged, suggesting that carbapenem resistance is emerging as the most dynamic concern within this species. Regarding methicillin-resistant and multidrug-resistant phenotypes, the prevalence of MRSP (27.4%) and MDR *S. pseudintermedius* (38.5%) did not change significantly between periods (*p* > 0.05). Similarly, no significant variation was observed for MDR *P. aeruginosa* isolates (53.3%) (*p* > 0.05) (Figure 7).

**Figure 8:**
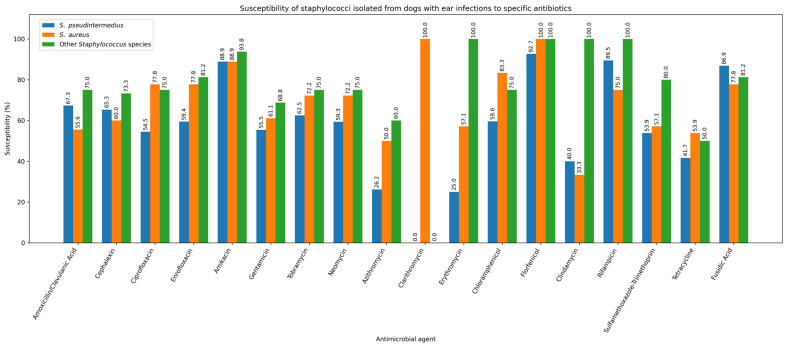
Susceptibility of staphylococci isolated from dogs with ear infections to specific antibiotics.

**Figure 9:**
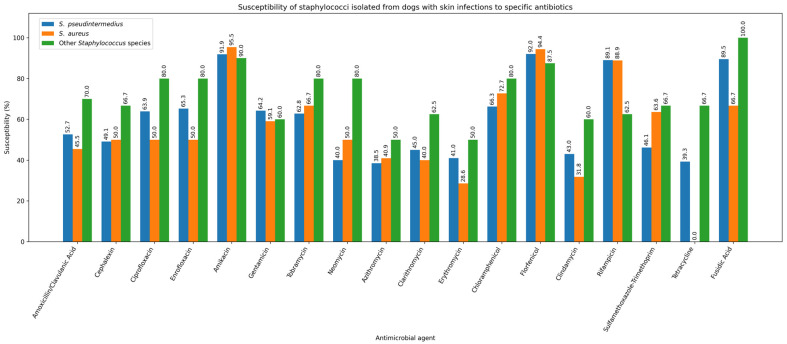
Susceptibility of staphylococci isolated from dogs with skin infections to specific antibiotics.

One strain of *S. pseudintermedius* (1/270; 0.37%) and one strain of *P. aeruginosa* (1/77; 1.30%) were resistant to all tested antibiotics. *S. pseudintermedius* was isolated from the skin, while *P. aeruginosa* was isolated from the external ear canal. Given that staphylococci are the predominant pathogens in canine skin and ear infections, Figure 8 and Figure 9 illustrate the antibiotic susceptibility profiles of staphylococcal isolates obtained from ear and skin samples.

## 3. Discussion

A thorough understanding of pathogen distribution, along with up-to-date antimicrobial susceptibility data, is essential for effectively managing OE and skin infections in both human and veterinary medicine [2]. In this study, *Staphylococcus* spp., *P. aeruginosa*, *Proteus* spp., and *Streptococcus* spp. were the most frequently isolated bacteria, which aligns with previous reports [1,6,7,8,9]. Our findings further confirm the role of *S. pseudintermedius* as the main causative agent of canine skin infections [1,2,8]. Other authors have also reported a high prevalence of this bacterium, ranging from 39.2% to 58.8% [1,10,11] for OE cases and 50.0% to 84.7% [12,13] for skin infection cases. The high rate of colonization with *Staphylococcus* spp. in dogs may pose a potential public health concern. For example, *S. coagulans* and *S. aureus* are associated with a broad spectrum of diseases in both humans and animals [14,15]. Although the detected low prevalence of *S. aureus* in our samples aligns with other available studies, their capacity to harbor antimicrobial resistance mechanisms underscores the importance of monitoring their role in canine infections and public health implications [2,16,17].

*P. aeruginosa* was the second most commonly isolated bacterium from canine ear samples, similarly to other reports [4,18]. By contrast, Bugden described this bacterium as the most frequently isolated species from canine OE samples in Australia, followed by *S. pseudintermedius*, linking the higher prevalence of *P. aeruginosa* to an increased diagnosis of chronic OE [19]. Although previous studies indicate low genetic similarity between canine and human *P. aeruginosa* strains, suggesting limited cross-species transmission, proper hygiene when handling animals remains important. This is supported by a 2018 report documenting zoonotic transmission of a carbapenem-resistant *P. aeruginosa* strain (VIM-2 producer) between a dog, its owner, and their household environment [20].

*Proteus* spp., especially *P. mirabilis*, were identified in our samples at a slightly higher frequency compared to other studies [1,2]. This finding underscores *Proteus* spp. as occasional yet noteworthy contributors to canine infections. Importantly, in all cases in this study, *Proteus* spp. was isolated as part of mixed infections, with at least one additional pathogen present, denoting their role as secondary pathogens. Their clinical relevance in this context lies primarily in the fact that mixed infections may complicate treatment outcomes, especially when empirical therapy lacks adequate Gram-negative coverage. Therefore, while the etiological importance of *Proteus* spp. is limited, their presence may contribute to persistence or recurrence of infection in predisposed cases [3,21]. High percentages of mixed infections, as noted in our study results, are clinically significant since they reduce the likelihood of successful treatment with empirical or antibiotic susceptibility-based therapy.

During the study period, ear and skin infections were predominantly observed in male dogs, similarly to some other reports [1,22]. However, sex distribution was assessed solely on a descriptive basis and was not intended to imply any causal association since the development of otitis is primarily influenced by other known factors [23].

Skin infections were most commonly diagnosed in dogs aged 5 to 10 years, consistent with findings from other studies [1]. In contrast, no statistically significant correlation between age and OE was observed. In younger dogs, otitis is frequently associated with ear mites (*Otodectes cynotis*) [24], while bacterial otitis is more prevalent in adult dogs. The latter often develops secondary to underlying conditions such as allergies, impaired immune function, endocrine disorders, or reduced elasticity of the skin and ear canal tissues, which predispose older dogs to bacterial infections [25]. However, some studies have reported a higher incidence of bacterial otitis in younger dogs, particularly those ≤4 years of age [18].

In our study, the West Highland White Terrier was the most common breed in otitis cases, followed by the Labrador Retriever, English Bulldog, English Cocker Spaniel, Golden Retriever, mixed breeds, and others. These findings align closely with other studies [2,18]. Although such a high incidence in the West Highland White Terrier has not been previously reported, it most likely reflects this breed’s predisposition to otitis [23] as well as its popularity in Serbia.

Antibiotic resistance has become a significant public health issue, largely driven by the improper use, overuse, and misuse of antibiotics in both human and veterinary medicine [26]. Conditions such as canine OE and bacterial skin infections are among the leading causes for prescribing antibiotics in small animal veterinary practices [1,27]. Despite the widespread use of antibiotics to treat these issues, antimicrobial susceptibility testing is rarely conducted, and treatments are often based on empirical practices rather than guided by sensitivity data [1,28]. The observed trend of increasing resistance of *S. pseudintermedius* to amoxicillin-clavulanate (AMC), cephalexin, and fusidic acid is not unexpected, given that broad-spectrum antibiotics are among the most frequently prescribed antimicrobials for dogs and cats [29]. Similary, data from Romania and North Macedonia similarly show β-lactam resistance in *S. pseudintermedius* ranging from 15.9% to 73% [30,31]. Furthermore, a study from Bulgaria noted a significant increase in resistance to AMC from 5 to 42% in coagulase-positive staphylococci from OE samples [32].

Although the prevalence of MRSP and MDR *S. pseudintermedius* did not show a statistically significant rise over the years, their rates remain alarmingly high. Neighboring countries report MRSP prevalence of 7.5% in Croatia [33] and 29% in Bosnia and Herzegovina [34], while our previous work also confirmed high MRSP prevalence in Serbia through *mecA* detection [8]. The elevated MRSP rate in this study may reflect the selection of OE and dermatitis cases, which frequently involve methicillin-resistant infections. MRSP and MDR strains with extensive resistance profiles represent a major challenge in veterinary medicine [35], and veterinary settings, such as hospitals and clinics, appear to be key hubs for their transmission between pets and humans [36].

A notable finding of this study was the persistently high resistance of *Staphylococcus* isolates to macrolide antibiotics and clindamycin throughout the entire study period. This pattern may reflect co-selection of erythromycin resistance during treatment with other antimicrobials. For example, the *ermB* gene is linked to the aadE-sat4-aphA-3 aminoglycoside-resistance cluster, meaning that use of topical aminoglycosides can inadvertently co-select for resistance to macrolides and lincosamides [37,38]. Consistent with a growing global trend of macrolide and clindamycin resistance, particularly among MRSP isolates, our findings align with ISCAID recommendations that prioritize topical therapy for canine OE and pyoderma and reserve systemic antibiotics such as cephalexin, cefadroxil, or AMC for appropriate cases, while advising against empirical use of macrolides and clindamycin in regions like ours where resistance to these classes is high [39].

Antibiotic resistance in the group ‘other staphylococci’ was significantly lower than in *S. aureus* and *S. pseudintermedius*, presumably due to the predominance of coagulase-negative staphylococci, which generally exhibit lower resistance levels compared to coagulase-positive strains [40]. However, higher resistance rates have been reported in *S. coagulans* [41], *S. haemolyticus* [42], and *S. epidermidis* [43]. Notably, amikacin and florfenicol emerged as two of the most effective antibiotics, with isolates showing high susceptibility, further supporting their clinical utility.

Regarding treatment efficacy, staphylococci demonstrated the highest sensitivity to amikacin, florfenicol, and rifampicin. Since OE is best managed with topical therapy [3] and neither amikacin nor rifampicin is commercially available in topical formulations, florfenicol appears most promising based on in vitro susceptibility data. Florfenicol, a synthetic derivative of thiamphenicol and chloramphenicol, inhibits bacterial protein synthesis and has documented activity against diverse Gram-positive and Gram-negative bacteria, with additional evidence suggesting anti-inflammatory properties [44]. It is also available in Serbia as an approved otic preparation for treating canine OE.

The highest resistance rates in β-hemolytic *Streptococcus* were observed against SXT and tetracycline, while AMC was among the most effective antibiotics, with isolates showing high susceptibility, supporting its classification as a drug of choice for treating canine streptococcal infections [21]. *Streptococcus* isolates in this study showed notably high levels of resistance to fluoroquinolones, with 53.66% resistant to ciprofloxacin and 51.22% to enrofloxacin. Earlier studies (2010–2016) reported good fluoroquinolone activity [45], but research from 2017–2025 demonstrates a consistent global increase, with *S. canis* resistance reaching 45–60% in canine OE cases across Europe, Asia, and Latin America [46,47]. This trend is largely linked to excessive empirical use of fluoroquinolones, particularly enrofloxacin and marbofloxacin, often without susceptibility testing. Despite this, Despite this, fluoroquinolones are prescribed in 63% of *S. canis* cases [47].

*P. aeruginosa* is intrinsically resistant to numerous antibiotics, including most β-lactams and β-lactamase inhibitor combinations, tetracycline, trimethoprim-sulfamethoxazole, and chloramphenicol, and readily develops additional resistance [1]. In our study, amikacin, polymyxin B, and imipenem showed the highest activity against *P. aeruginosa*. Imipenem resistance was low, slightly below the 15–23% reported elsewhere [48,49], although still clinically relevant given WHO’s designation of carbapenem-resistant *P. aeruginosa* as a critical priority pathogen [50]. The significant resistance of *P. aeruginosa* isolates from canine OE to gentamicin (19.32%) observed in this study aligns with the results reported in previous research [4,51]. Petrov et al. noted the decrease in resistance rates for AMC, while a significant increase was detected for aminoglycosides [32]. An earlier study from Croatia describes *P. aeruginosa* strain resistance to gentamicin (43.3%), ciprofloxacin (8.7%), and enrofloxacin (51.9%) [51]. This study did not include several key anti-pseudomonal agents (e.g., piperacillin-tazobactam, ceftazidime, aztreonam), limiting the scope of susceptibility data. Notably, *P. aeruginosa* showed the highest proportion of MDR strains among all tested bacteria, greatly restricting therapeutic options, as reported elsewhere [1,52].

The high resistance observed for AMC as well as to first-generation cephalosporins (cephalexin) across all isolates except *Streptococcus* spp. are likely influenced by the fact that, besides clindamycin, these antibiotics are commonly used as first-line treatments during empirical antibiotic prescription, contributing to the emergence of AMR [2,39]. Also, of concern is the fact that we detected variable (fluctuating) levels of resistance to fluoroquinolones given that they are classified as critically important antibiotics in human medicine [53,54]. A striking finding was the high level of resistance to tetracycline across all tested bacterial species, and it should be regarded as a poor empirical choice for the treatment of ear and skin infections in dogs, consistent with several recent reports describing similar resistance levels [30,55,56].

The prevalence of MDR strains in our examination corresponds to findings from recent reports from studies on dogs with otitis and skin infections [4,57,58]. MDR pathogens pose a significant threat to animal health by severely limiting therapeutic options and often necessitating the use of antibiotics not approved for veterinary use. These findings highlight the need to establish treatment protocols for managing canine OE and skin infections, tailored and informed by the latest research on resistance profiles of commonly isolated pathogens, as well as continuous AMR monitoring through national programs like the German GERM-Vet, the Swedish SVARM, and the French RESAPATH, which are already established in some European countries [59,60,61]. According to the Statistical Office of the Republic of Serbia, the country has an estimated population of approximately 6.59 million inhabitants [62], while data from the Veterinary Directorate of the Republic of Serbia indicates that 1,955,289 dogs are registered in the Central Database. Additionally, around 100,000 new dogs are registered annually, and between 30,000 and 35,000 are deregistered, either due to death or export [63]. This information underscores the importance of implementing the mentioned protocols and monitoring systems in Serbia. This retrospective study provides valuable insights for clinicians, particularly in veterinary hospitals within our region and neighboring countries, to make informed decisions regarding antibiotic use, especially considering that this is the first study of its kind conducted in Serbia. Because empirical antibiotic use is still common in everyday veterinary practice, and studies show that evidence-based prescribing is often overlooked, regular susceptibility testing has become increasingly important for choosing effective treatments, preventing resistance, and improving patient outcomes [1,2]. This need is even more pronounced in recurrent cases, and our findings reinforce how essential it is to keep evaluating the bacteria involved in canine ear and skin infections so that treatment decisions remain well-informed and clinically sound. Compared to published Balkan studies that report only single-timepoint prevalence, or class-specific resistance rates, this study is the first from Serbia to offer a large-scale longitudinal analysis with statistically evaluated AMR trends for major canine skin and ear pathogens.

## 4. Materials and Methods

### 4.1. Data Collection

This study analyzed data from the Laboratory for Clinical Bacteriology and Mycology at the Department of Microbiology and Dermatological Clinic, FVM, University of Belgrade, Serbia, covering January 2017 to August 2024. The dataset, collected from VetIS (Veterinary Information System, a nationally developed digital platform created by the Computer Centre of the Faculty of Electrical Engineering, University of Belgrade), which enables recording and tracking of veterinary data, including diagnostics, treatments, and laboratory findings, consisted of records from clinically ill canine patients diagnosed with OE and pyoderma or postsurgical wound infections at the Dermatology Clinic of the Faculty of Veterinary Medicine. A total of 422 noninvasive skin and ear swab samples were collected during routine veterinary examinations conducted for diagnostic purposes. The laboratory records included essential information, such as animal identification details (name, ID number, species, breed, sex, and age), type of material collected, clinical diagnosis, sampling date, prior antibiotic use, bacterial identification, and antimicrobial susceptibility testing results. For statistical analysis, age was categorized into three groups (<5 years, 5–10 years, and >10 years). Additionally, co-infections in positive cultures were recorded (excluding fungi). Collected data were organized in Microsoft Excel before statistical analysis to ensure proper standardization. Incomplete or missing records were excluded from the study to maintain data integrity. All variables were clearly defined and cross-verified with laboratory logs to establish accuracy.

### 4.2. Isolation and Identification of Bacteria and Antimicrobial Susceptibility Testing

For microbiological analysis, the samples were cultured using the following media: Columbia agar with 5% sheep blood (Becton Dickinson, Franklin Lakes, NJ, USA), MacConkey agar (Becton Dickinson, Franklin Lakes, NJ, USA), and Sabouraud dextrose agar (HiMedia, Mumbai, India) for fungal isolation, though fungi were excluded from this study. For the rapid identification of Enterobacterales, the chromogenic HICrome UTI agar (HiMedia, Mumbai, India) was used. Conventional clinical microbiology tests were used to establish an accurate identification (Gram staining, catalase, oxidase, assessment of beta-hemolysis, biochemical properties such as indole utilization, etc.). Additional tests included the ONPG (β-galactosidase) test, mannitol test, urease test, polymyxin E susceptibility testing, and coagulase tests for differentiating staphylococci. Staphylococci were identified using the commercial ID 32 Staph system (API, BioMérieux, Lyon, France), streptococci were classified into groups using specific agglutinating sera (Microgen Bioproducts Ltd., Camberley, UK), and for Gram-negative bacteria the BBL Crystal Enteric/Nonfermentor Kit (Becton Dickinson, Franklin Lakes, NJ, USA) was used.

This study analyzed the antibiotic resistance profiles of *S. pseudintermedius*, *S. aureus*, other staphylococci (grouped), *Streptococcus* Lancefield group G (presumptive *S. canis*), *P. aeruginosa* and *P. mirabilis*. Antibiotic resistance profiles of other strains were excluded from the analysis due to the limited number of isolates. Antimicrobial susceptibility testing was performed on Mueller-Hinton agar (Becton Dickinson, Heidelberg, Germany) using the disk diffusion method, following CLSI and EUCAST guidelines. For staphylococci, antimicrobial susceptibility was interpreted according to CLSI VET01-A4 [64] and updated annually using CLSI M100 documents [65,66,67,68,69,70,71,72] for agents not covered by VET01-A4 and EUCAST guedelines [73,74,75,76,77,78,79,80]. For β-hemolytic streptococci, EUCAST streptococcal clinical breakpoints (versions 2017–2024) [73,74,75,76,77,78,79,80] were applied whenever available, while CLSI VET01-A4 [64] and CLSI M100 [65,66,67,68,69,70,71,72] were used for antibiotics lacking EUCAST values. For *P. aeruginosa* and *P. mirabilis*, EUCAST clinical breakpoints (versions 2017–2024) [73,74,75,76,77,78,79,80] were applied for all antibiotics included in EUCAST tables. For agents lacking EUCAST interpretation, particularly those used primarily in veterinary medicine, CLSI M100 [65,66,67,68,69,70,71,72] and CLSI VET01-A4 [64] were used. Breakpoints were updated annually throughout the 8-year study period according to the most recent documents available at the time of testing.

The results should therefore be interpreted with caution. A total of 20 antimicrobial agents were tested (Becton Dickinson, Franklin Lakes, NJ, USA (except for the florfenicol, purchased from Oxoid, Basingstoke, UK) using the following concentrations: Amoxicillin/Clavulanic acid 20 μg + 10 μg (AMC), Cefalexin 30 μg (CN), Amikacin 30 μg (AN), Gentamicin 10 μg (GM), Tobramycin 10 μg (NN), Neomycin 30 μg (N), Ciprofloxacin 5 μg (CIP), Enrofloxacin 5 μg (ENF), Erythromycin 15 μg (E), Azithromycin 15 μg (AZM), Clarithromycin 15 μg (CLR), Chloramphenicol 30 μg (C), Florfenicol 30 μg (FFC), Clindamycin 2 μg (CC), Rifampicin 5 μg (RAM), Fusidic acid 10 μg (FA), Trimethoprim/Sulfamethoxazole 1.25 μg + 23.75 μg (SXT), Tetracycline 30 μg (TET), Imipenem 10 μg (IPM) and Polymyxin B 300 IU (PB). Due to the duration of this study and the specific characteristics of certain isolates and clinical cases, as well as considering intrinsic resistance [81] and official recommendations, not all isolates were consistently tested against every mentioned antibiotic. The number of tested isolates is provided in the study results (Table 2). Quality control of each new lot of antibiotic disks and culture media was performed according to CLSI VET01-A4 [64] and CLSI M100 [65,66,67,68,69,70,71,72]. The potency and integrity of disks, as well as the sterility, depth, and pH of Mueller–Hinton agar, were verified before testing. Reference strains *S. aureus* ATCC 25923, *E. coli* ATCC 25922, *P. aeruginosa* ATCC 27853, and *E. faecalis* ATCC 29212 (Microbiologics, St. Cloud, MN, USA) were used for routine performance checks.

Methicillin resistance in staphylococci was evaluated using the disc diffusion method with cefoxitin (30 μg) (Becton Dickinson, USA) for S. aureus and oxacillin (2 μg) (Becton Dickinson, Franklin Lakes, NJ, USA) for other staphylococci [64,82,83]. Confirmation of MRSA was done by PCR using MRSA ATCC 43300 and ATCC 33591 (Microbiologics, St. Cloud, MN, USA) as positive controls, with the previously described method [84]. The *mecC* gene was not tested. Also, detection of PBP2a was done with the Slidex MRSA agglutination kit (bioMérieux, Lyon, France). Multidrug resistance (MDR) was defined as resistance to at least three distinct antibiotic classes [85]. The considered antibiotic classes were as follows: cephalosporins, fluoroquinolones, aminoglycosides, tetracycline, macrolides and amphenicoles.

### 4.3. Data Management and Statistical Analysis

Descriptive statistics were used to analyze the proportions of each group, including age, sex, skin infection types, and antimicrobial drugs. Graphics were generated using R’s built-in libraries, and the frequencies of antimicrobial resistance were summarized through descriptive statistical analysis. The statistical significance between groups was evaluated using Fisher’s exact test, with a *p*-value < 0.05 considered significant. Statistical analysis of the experimental results was performed using the statistical software GraphPad Prism version 6 (GraphPad, San Diego, CA, USA).

## 5. Conclusions

Throughout the study period, *S. pseudintermedius* and *P. aeruginosa* were identified as the predominant pathogens in canine skin and ear infections. A clear upward trend in antimicrobial resistance among *S. pseudintermedius* isolates was observed for β-lactams, particularly amoxicillin–clavulanate and cephalexin, followed by fusidic acid and amikacin. Florfenicol demonstrated a decreasing resistance trend and remained highly effective for localized treatment of staphylococcal otitis. In contrast, tetracyclines exhibited persistently high resistance across all bacterial species, making them a poor therapeutic choice for both skin and ear infections. Resistance to macrolides and clindamycin remained consistently high throughout the period without notable fluctuation. In *P. aeruginosa*, a significant upward trend in resistance to imipenem was recorded in the later study years, while susceptibility to amikacin and polymyxin B remained stable. The persistently high prevalence of methicillin-resistant and multidrug-resistant *S. pseudintermedius* and *P. aeruginosa* continues to limit therapeutic options. These findings highlight the growing challenge of antimicrobial resistance in small-animal practice and the importance of routine susceptibility testing to guide rational antibiotic selection.

## Figures and Tables

**Table 1:** Bacterial isolates from canine ear and skin infections.

Isolated Bacteria	Ears		Skin	
	*n*	%	*n*	%
***S. pseudintermedius***	101	48.10	169	79.72
***S. aureus***	18	8.57	22	10.38
**Other staphylococci**	16	7.62	10	4.72
*S. coagulans*	1	0.48	4	1.89
*S. haemolyticus*	3	1.43	2	0.94
*S. epidermidis*	12	5.71	4	1.89
β-haemolytic *Streptococcus* group G	23	10.59	18	8.49
*Pseudomonas aeruginosa*	61	29.05	16	7.55
***Proteus* spp.**	39	18.57	18	8.49
*P. vulgaris*	9	4.29	8	3.77
*P. mirabilis*	30	14.29	10	4.72
***Enterobacterales***	9	4.29	11	5.19
*Escherichia coli*	4	1.90	11	5.19
*Klebsiella pneumoniae*	4	1.90	0	0.00
*Serratia marcescens*	1	0.48	0	0.00
**Other bacteria**	20	9.52	6	2.83
*Corynebacterium* sp.	17	8.10	3	1.42
*Pseudomonas* sp. (other than *P. aeruginosa*)	1	0.48	2	0.94

**Table 2:** Distribution of antimicrobial susceptibility profiles among the most prevalent bacterial pathogens.

Drug	*S. pseudintermedius*		*S. aureus*		Other Staphylococci		β-Hemolytic *Streptococcus* (Group G)		*P. aeruginosa*		*P. mirabilis*	
	S	R	S	R	S	R	S	R	S	R	S	R
AMC	58.15% (*n* = 157)	41.85% (*n* = 113)	50.00% (*n* = 20)	50.00% (*n* = 20)	73.0% (*n* = 19)	26.92% (*n* = 7)	90.24% (*n* = 37)	9.76% (*n* = 4)	/	/	72.50% (*n* = 29)	27.50% (*n* = 11)
CN	55.30% (*n* = 146)	44.70% (*n* = 118)	53.13% (*n* = 17)	46.88% (*n* = 15)	70.83% (*n* = 17)	29.17% (*n* = 7)	92.31% (*n* = 36)	7.69% (*n* = 3)	/	/	70.00% (*n* = 28)	30.00% (*n* = 12)
CIP	60.37% (*n* = 163)	39.63% (*n* = 107)	62.50% (*n* = 25)	37.50% (*n* = 15)	76.92% (*n* = 20)	23.08% (*n* = 6)	46.34% (*n* = 19)	53.66% (*n* = 22)	81.82% (*n* = 63)	18.18% (*n* = 14)	95.00% (*n* = 38)	5.00% (*n* = 2)
ENF	63.06% (*n* = 169)	36.94% (*n* = 99)	62.50% (*n* = 25)	37.50% (*n* = 15)	80.77% (*n* = 21)	19.23% (*n* = 5)	48.78% (*n* = 20)	51.22% (*n* = 21)	83.12% (*n* = 64)	16.88% (*n* = 13)	95.00% (*n* = 38)	5.00% (*n* = 2)
AN	90.73% (*n* = 235)	9.27% (*n* = 24)	92.50% (*n* = 37)	7.50% (*n* = 3)	92.31% (*n* = 24)	7.69% (*n* = 2)	/	/	90.79% (*n* = 69)	9.21% (*n* = 7)	94.87% (*n* = 37)	5.13% (*n* = 2)
GM	60.90% (*n* = 162)	39.10% (*n* = 104)	60.00% (*n* = 24)	40.00% (*n* = 16)	65.38% (*n* = 17)	34.62% (*n* = 9)	/	/	80.68% (*n* = 71)	19.32% (*n* = 17)	90.00% (*n* = 36)	10.00% (*n* = 4)
NN	62.64% (*n* = 109)	37.36% (*n* = 65)	71.43% (*n* = 15)	28.57% (*n* = 6)	76.19% (*n* = 16)	23.81% (*n* = 5)	/	/	80.56% (*n* = 58)	19.44% (*n* = 14)	90.00% (*n* = 36)	10.00% (*n* = 4)
N	57.43% (*n* = 58)	42.57% (*n* = 43)	70.00% (*n* = 14)	30.00% (*n* = 6)	76.19% (*n* = 16)	23.81% (*n* = 5)	/	/	/	/	77.50% (*n* = 31)	22.50% (*n* = 9)
AZM	35.69% (*n* = 73)	64.04% (*n* = 130)	41.67% (*n* = 10)	58.33% (*n* = 14)	53.33% (*n* = 8)	46.67% (*n* = 7)	81.08% (*n* = 30)	18.92% (*n* = 7)	/	/	/	/
CLR	41.86% (*n* = 18)	58.14% (*n* = 25)	43.75% (*n* = 7)	56.25% (*n* = 9)	/	/	83.33% (*n* = 5)	16.67% (*n* = 1)	/	/	/	/
E	37.25% (*n* = 57)	62.75% (*n* = 96)	42.86% (*n* = 6)	57.14% (*n* = 8)	60.00% (*n* = 3)	40.00% (*n* = 2)	73.08% (*n* = 19)	26.92% (*n* = 7)	/	/	/	/
C	63.74% (*n* = 167)	36.26% (*n* = 95)	77.50% (*n* = 31)	22.50% (*n* = 9)	76.92% (*n* = 20)	23.08% (*n* = 6)	92.68% (*n* = 38)	7.32% (*n* = 3)	/	/	64.10% (*n* = 25)	35.90% (*n* = 14)
FFC	92.35% (*n* = 157)	7.65% (*n* = 13)	97.22% (*n* = 35)	2.78% (*n* = 1)	95.83% (*n* = 23)	4.17% (*n* = 1)	96.43% (*n* = 27)	3.57% (*n* = 1)	49.15% (*n* = 29)	50.85% (*n* = 30)	70.27% (*n* = 26)	29.73% (*n* = 11)
CC	42.33% (*n* = 91)	57.67% (*n* = 124)	32.00% (*n* = 8)	68.00% (*n* = 17)	63.64% (*n* = 7)	36.36% (*n* = 4)	70.59% (*n* = 24)	29.41% (*n* = 10)	/	/	/	/
RAM	89.16% (*n* = 148)	10.84% (*n* = 18)	86.36% (*n* = 19)	13.64% (*n* = 3)	66.67% (*n* = 6)	33.33% (*n* = 3)	92.31% (*n* = 12)	7.69% (*n* = 1)	/	/	/	/
SXT	48.29% (*n* = 113)	51.71% (*n* = 121)	62.07% (*n* = 18)	37.93% (*n* = 11)	71.43% (*n* = 10)	28.57% (*n* = 4)	34.38% (*n* = 11)	65.63% (*n* = 21)	/	/	51.28% (*n* = 20)	48.72% (*n* = 19)
TET	40.38% (*n* = 63)	59.62% (*n* = 93)	41.18% (*n* = 7)	58.82% (*n* = 10)	56.25% (*n* = 9)	43.75% (*n* = 7)	25.00% (*n* = 4)	75.00% (*n* = 12)	/	/	52.50% (*n* = 21)	47.50% (*n* = 19)
FA	87.95% (*n* = 146)	12.05% (*n* = 20)	76.19% (*n* = 16)	23.81% (*n* = 5)	82.35% (*n* = 14)	17.65% (*n* = 3)	35.71% (*n* = 10)	64.29% (*n* = 18)	/	/	/	/
PB	/	/	/	/	/	/	/	/	89.55% (*n* = 60)	10.45% (*n* = 7)	/	/
IMP	/	/	/	/	/	/	/	/	88.89% (*n* = 48)	11.11% (*n* = 6)	100.00% (*n* = 26)	0.00% (*n* = 0)

/—Antibiotic has not been tested. Legend: AMC—Amoxicillin/Clavulanic acid, CN—Cephalexin, AN—Amikacin, GM—Gentamicin, NN—Tobramycin, N—Neomycin, CIP—Ciprofloxacin, ENF—Enrofloxacin, E—Erythromycin, AZM—Azithromycin, CLR—Clarithromycin, C—Chloramphenicol, FFC—Florfenicol, CC—Clindamycin, RAM—Rifampicin, FA—Fusidic acid, SXT—Trimethoprim/Sulfamethoxazole, TET—Tetracycline, IMP—Imipenem, PB—Polymyxin B.

**Table 3:** The percentages of methicillin-resistant staphylococci (MRS) and multidrug-resistant (MDR) bacteria during the 2017–2024 period.

Category	Positive Isolates *n* (%)	Total Number of Tested Isolates (*n*)
MRSP	74 (27.41%)	270
MRSA	9 (22.50%)	40
Methicillin-resistance in other staphylococci	2 (7.69%)	26
MDR *S. pseudintermedius*	104 (38.52%)	270
MDR *S. aureus*	11 (27.50%)	40
MDR—other staphylococci	2 (7.69%)	26
MDR β-hemolytic *Streptococcus* (group G)	14 (34.15%)	41
MDR *Proteus mirabilis*	19 (33.33%)	57
MDR *P. aeruginosa*	41 (53.25%)	77

MRSP—Methicillin-resistant *S. pseudintermedius*; MRSA—Methicillin-resistant *S. aureus*; MDR—Multidrug resistant. Methicillin-resistance (MRSP) was analyzed separately from multidrug resistance (MDR), as not all methicillin-resistant isolates meet the MDR definition (resistance to ≥3 antimicrobial classes). However, overlap between MRSP and MDR isolates was observed.

## Data Availability

The data presented in this study are available on request from the corresponding author. The data are not publicly available due to veterinary privacy regulations and ethical restrictions.

## Abbreviations

The following abbreviations are used in this manuscript:

AMC	Amoxicillin/Clavulanic acid
CN	Cephalexin
AN	Amikacin
GM	Gentamicin
NN	Tobramycin
N	Neomycin
CIP	Ciprofloxacin
ENF	Enrofloxacin
E	Erythromycin
AZM	Azithromycin
CLR	Clarithromycin
C	Chloramphenicol
FFC	Florfenicol
C	Clindamycin
RAM	Rifampicin
FA	Fusidic acid
SXT	Trimethoprim/Sulfamethoxazole
TET	Tetracycline
IMP	Imipenem
PB	Polymyxin B
MRSP	Methicillin-resistant *S. pseudintermedius*
MRSA	Methicillin-resistant *S. aureus*
MDR	Multidrug resistant
